# Emerging Insights into the Liver–Pancreas Axis: A Central Hub in the Pathogenesis of Diabetes and Metabolic Diseases

**DOI:** 10.3390/biom16040613

**Published:** 2026-04-21

**Authors:** Hengqian Dai, Ziyi Zhang

**Affiliations:** Department of Endocrinology, Sir Run Run Shaw Hospital, Zhejiang University School of Medicine, Hangzhou 310016, China; 22418529@zju.edu.cn

**Keywords:** metabolic dysfunction-associated fatty liver disease, type 2 diabetes, liver–pancreas axis, lipid metabolism, glucose metabolism

## Abstract

Diabetes and related metabolic disorders, including metabolic dysfunction-associated steatotic liver disease (MASLD), are increasingly recognized as diseases of inter-organ metabolic dysregulation rather than disorders of a single organ. The core of this process is the liver–pancreas axis, which integrates metabolic signals to maintain glucose and lipid homeostasis. Under physiological conditions, insulin and glucagon work together to regulate glucose production in the liver. The liver, in turn, regulates pancreatic β-cell function through hepatokines, metabolites and extracellular vesicles. Axis disorder driven by liver insulin resistance, lipid accumulation, inflammation or changes in hepatokine secretion exacerbates β-cell dysfunction, glucotoxicity and lipotoxic stress, thereby accelerating disease progression. This imbalance is involved in the pathogenesis of type 2 diabetes, type 1 diabetes, gestational diabetes, and monogenic diabetes, and makes MASLD a driving factor and early predictor of diabetes onset. This review summarizes the key molecular mechanisms behind liver–pancreas crosstalk and explores potential therapeutic strategies aimed at restoring coordinated metabolic regulation between the organs.

## 1. Introduction

Diabetes and other metabolic diseases, such as obesity and metabolic dysfunction-associated steatotic liver disease (MASLD) [1], have become major global health problems and are some of the most severe public health challenges nowadays [2,3,4,5]. Between 1990 and 2021, the number of adult diabetes patients worldwide more than doubled, with type 2 diabetes (T2D) accounting for over 90% of all cases [2]. At the same time, diabetes-related complications (including cardiovascular disease, renal failure and hepatic steatosis) have greatly exacerbated disability and mortality worldwide [6,7,8]. In addition, diabetes can significantly reduce patients’ quality of life, bringing a huge medical and economic burden to countries and regions [9].

Although diabetes has traditionally been regarded as a disease caused by simple pancreatic β-cell failure or peripheral insulin resistance, an increasing amount of evidence indicates that diabetes and metabolic syndrome are caused by overall dysfunction of multiple metabolic organs, especially the liver and pancreas [10,11]. The concept of a liver–pancreas axis emerged gradually as studies identified liver-derived endocrine factors and metabolic signals that modulate peripheral insulin sensitivity and islet function [12,13]. Physiologically, the pancreas secretes insulin and glucagon to modulate hepatic glucose production, glycogen storage, and lipid synthesis, while the liver in turn influences pancreatic function via hepatokines (e.g., fibroblast growth factor 21 (FGF21), fetuin-A) and metabolites that regulate β-cell growth, insulin secretion and survival [14,15,16,17,18,19].

Disruption of this axis such as by hepatic insulin resistance, lipid accumulation, or inflammation can impair β-cell function, leading to hyperinsulinemia, glucotoxicity, and eventual β-cell exhaustion [20,21,22,23], and is involved in the pathogenesis of various metabolic diseases. Although type 1 diabetes (T1D) is classically characterized by a reduction in autoimmune β-cell and insulin deficiency, the liver–pancreas axis in patients with T1D undergoes significant changes, leading to excessive liver glucose production and dyslipidemia [20,24]. In T2D, abnormal liver metabolism and pancreatic β-cell dysfunction form a vicious cycle, accelerating disease progression [25,26]. During gestational diabetes mellitus (GDM), hepatic metabolic reprogramming and changes in pancreatic insulin secretion are closely related, which jointly affect the glucose homeostasis of both mother and fetus [27]. In MASLD, lipid overload in the liver can trigger lipid toxicity and inflammatory responses, thereby damage the integrity and function of pancreatic β cells [23,28,29,30].

However, current studies often focus on hepatic or pancreatic dysfunction in isolation and ignore the bidirectionality and dynamics of their interactions, particularly the critical role of endocrine mediators in inter-organ communication. In this review, we highlight the key distinctions from prior studies by providing a systematic and integrated framework of bidirectional liver–pancreas crosstalk, with endocrine factors as central mediators. We further incorporate emerging mediators (e.g., extracellular vesicles, miRNAs, bile acids, and branched-chain amino acids [BCAAs]), extend the axis concept across multiple diabetes types (T1D, T2D, GDM, and monogenic diabetes), integrate multi-omics approaches, and position MASLD as an early predictor of diabetes, while outlining future directions toward phenotype-driven precision medicine.

## 2. Physiological Crosstalk Between Liver and Pancreas

### 2.1. Insulin, Glucagon, and Hepatic Glucose Metabolism

As the core link in the regulation of the liver–pancreas axis, insulin and glucagon jointly coordinate the production and utilization of glucose in the liver, thereby maintaining glucose homeostasis. These two hormones also regulate liver cell metabolism through different signaling pathways, which is particularly crucial for the blood glucose control.

In hepatocytes, insulin binds to insulin receptors (IRs) and activates the PI3K-AKT signaling pathway, leading to phosphorylation of the downstream factors that regulate glucose metabolism. Activated AKT promotes glycogen synthesis by stimulating glycogen synthase, while inhibiting gluconeogenesis by phosphorylation and inactivation of transcription factor forkhead box O1 (FOXO1). The silence of FOXO1 will reduce the expression of key gluconeogenesis enzymes, including phosphoenolpyruvate carboxykinase (PEPCK) and glucose-6-phosphatase (G6Pase), while enhancing glycolysis and glucose utilization [31,32,33]. However, when hepatic insulin resistance occurs, the PI3K-AKT-FOXO1 pathway is damaged, making it hard to fully inhibit glycogenesis, resulting in excessive liver glucose output and hyperglycemia. The resulting hyperglycemia can stimulate compensatory hyperinsulinemia and β-cell overload, further exacerbating the metabolic imbalance in T2D and MASLD [34,35].

Unlike insulin, glucagon maintains blood glucose level by promoting glucose production during fasting. After binding to G protein-coupled receptors (GPCRs) in hepatocytes, glucagon activates adenylate cyclase (AC), increases the intracellular level of cyclic adenosine monophosphate (cAMP), and activates protein kinase A (PKA). Activated PKA therefore promotes glycogen phosphorylase activity to accelerate glycogenolysis and enhances gluconeogenesis through the cyclic AMP-responsive element-binding protein (CREB), CREB-regulated transcription coactivator 2 (CRTC2), and peroxisome proliferator-activated receptor γ co-activator 1α (PGC-1α) transcriptional network, thereby increasing glucose output in the liver [36,37,38,39]. However, in T2D and MASLD, glucagon signaling often becomes disordered, manifested as decreased glucagon receptor (GCGR) sensitivity but continuous increase in circulating glucagon, thereby further driving fasting hyperglycemia and systemic metabolic imbalance [40,41].

### 2.2. Hepatokines and Pancreatic Feedback Regulation

A variety of hepatokines have been identified as important mediators of the liver–pancreas axis. Key hepatokines involved in liver–pancreas axis signaling and their effects on pancreatic β-cell function are summarized in Table 1. Among these mediators, multiple liver factors (fetuin-A, FGF21, ANGPTL8, SeP and IGF-1) act as key molecular signals in the liver and affect the function of the pancreas.

Fetuin-A, a glycoprotein secreted by the liver, not only interferes with the insulin receptor signals of β cells and peripheral tissues, but also induces inflammation and insulin resistance [42,43]. Clinically, the levels of circulating fetuin-A are elevated in patients with MASLD, T2D, insulin resistance and obesity, and are associated with hepatic steatosis, inflammatory markers and a reduction in the number of β cells [18,43,44]. An elevated level of fetuin-A exerts its effects through two main pathways: first, it binds to the Toll-like receptor 4 (TLR4) complex, activating the NF-κB/JNK-mediated inflammatory cascade in adipose tissue, liver and islets, promoting insulin resistance and β-cell dysfunction [43,45,46]; second, it disrupts the TGFβ receptor–SMAD2/3 axis in β cells, thereby damaging the maturation and compensatory proliferation of β cells [18]. Fetuin-A also inhibits the autophosphorylation of insulin receptor tyrosine kinase, weakens PI3K-AKT signaling, and antagonizes adiponectin, an adipokine with insulin-sensitizing effects [47]. Fetuin-A plays the role of a pathogenic amplifier, directly linking liver metabolic stress with pancreatic β-cell dysfunction.

In contrast, FGF21, secreted by hepatocytes, activates downstream signal transduction in target tissues by binding to the β-Klotho/fibroblast growth factor receptor (FGFR) complex to form a trimer receptor assembly [48,49]. In hepatocytes, FGF21 exerts its influence through the PPARα-FGF21-PGC-1α signaling pathway, promoting fatty acid oxidation, improving mitochondrial function and enhancing glucose utilization, thereby alleviating glucolipotoxic stress in β cells [50,51]. FGF21 also has a protective effect on islet function and maintaining β-cell survival. In β cells, FGF21 can activate the Akt/BAD and ERK1/2 pathways, exerting a dual cytoprotective effect by preventing apoptosis and enhancing insulin biosynthesis [17,48]. Under conditions of T2D and chronic metabolic stress, the expression and function of the β-Klotho/FGFR complex are often downregulated, leading to impaired FGF21 signal transduction [49].

ANGPTL8 (betatrophin) is a nutritional-responsive molecule that mainly works in coordination with ANGPTL3 to regulate the activity of lipoprotein lipase (LPL) and redistribute triglyceride (TAG) between tissues [52]. This lipid distribution function makes ANGPTL8 a key regulator of lipid flow within the liver–pancreas axis. Initial studies, mainly based on mouse models, suggested that ANGPTL8 may promote β-cell proliferation; however, subsequent investigations, including independent animal studies, have failed to consistently reproduce this effect, and human studies have not provided supportive evidence for a role in β-cell expansion [53,54]. Accordingly, current evidence supports that ANGPTL8 predominantly functions in lipid metabolism rather than as a direct regulator of β-cell proliferation. At the molecular level, mechanistic studies in hepatocytes and *in vivo* models have demonstrated that ANGPTL8 inhibits gluconeogenesis by regulating pathways including Akt-related metabolic signals and regulates energy metabolism in hepatocytes [55]. Therefore, its impact on β-cell function is largely indirect, through lipid redistribution and regulation of metabolic load rather than direct regulation of β cells. The exact β-cell-specific effect still requires further mechanism clarification.

Selenoprotein P (SeP), encoded by SEPP1, is regarded as a causal mediator of insulin resistance and β-cell dysfunction. Mechanism studies have shown that overexpression of SeP in the liver induces insulin resistance by inhibiting adenosine monophosphate-activated protein kinase (AMPK) signaling and regulating FOXO transcriptional activity, thereby linking the alteration of liver SeP production to the disruption of systemic glucose homeostasis [56]. SeP can also disrupt the redox balance within the target tissue, promoting a reductive oxidative stress environment, thereby impairing β-cell function and insulin secretion [57]. Clinically, the circulating SeP levels in obese, MASLD and T2D patients are elevated and are associated with indices of insulin resistance and glycemic control [58]. Importantly, antidiabetic drugs such as metformin downregulate the expression of SEPP1 in the liver through the AMPK-FOXO pathway, which reveals the mechanism link between existing treatment methods and SeP biology [58,59].

Insulin-like growth factor 1 (IGF-1) is mainly produced by the liver under the regulation of growth hormones (GHs). By activating the insulin-like growth factor 1 receptor (IGF1R) in β cells and peripheral tissues, IGF-1 initiates the downstream IRS-PI3K-AKT and RAS-Raf-MEK-ERK pathways. This promotes the survival of β cells, insulin biosynthesis and insulin secretion stimulated by glucose, while inhibiting the pro-apoptotic program [60,61]. IGF-1 produced by the liver can also regulate systemic insulin sensitivity and interact with the GH-IGF axis, affecting hepatic gluconeogenesis and lipid processing [62]. Under the state of chronic metabolic stress, the cross-interaction with insulin signaling and the compensatory changes at downstream nodes will reprogram the reactivity of IGF-1, which promotes the transformation of β cells from adaptive compensation to functional decline during the progression of metabolic diseases [63,64].

Other pancreatic peptides, such as amylin and pancreatic polypeptide (PP), are also involved in hepatic metabolic regulation. Amylin reduces postprandial glucagon secretion and inhibits hepatic glucose production; accordingly, the human amylin analog pramlintide, when used in combination with insulin, improves blood glucose control [65]; meanwhile, PP regulates appetite and liver glucose output through the central and vagus nerves [66], adding more neuroendocrine regulation to the liver–pancreas axis. These hormones work together to coordinate the nutrient flow between the pancreas and the liver to maintain glucose homeostasis throughout the body.

### 2.3. Role of Metabolites, Incretins, and Bile Acids

The regulation of the liver–pancreas axis is not limited to the effects of protein-based hormones and hepatokines. A variety of metabolites, bile acids, and incretins constitute an important network connecting the liver and the pancreas. These endocrine factors are also involved in the regulation of pancreatic islet cell function, glucose metabolism and lipid processing.

Recent studies highlight that it is not the total hepatic TAG content but rather specific lipid species, particularly diacylglycerols (DAGs, especially membrane-associated sn-1, 2-DAG) and sphingolipids/ceramides, that play pivotal roles in dysregulation of the liver–pancreas axis. Accumulation of membrane-localized DAG is a key driver of hepatic insulin resistance: membrane DAG activates PKCε, which inhibits the tyrosine kinase activity of IRs, thereby impairing downstream IRS-PI3K-Akt signaling pathway and weakening insulin suppression of gluconeogenesis and glycogen metabolism [67,68,69]. In parallel, ceramides have been widely implicated in hepatic insulin resistance and MASLD progression. Ceramides can activate atypical PKCζ or the phosphatase PP2A, reducing Akt translocation and phosphorylation, while also engaging stress pathways such as JNK to promote inhibitory serine phosphorylation of IRS-1 [70,71]. Elevated hepatic or circulating long-chain saturated ceramides (e.g., C16/C18) are consistently associated with impaired insulin sensitivity, hepatic steatosis, and pancreatic β-cell apoptosis [72,73].

BCAAs play a dual role in the liver–pancreas axis: they are not only important substrates supporting liver protein synthesis, energy metabolism and pancreatic hormone production, but also act as signaling molecules, precisely regulating liver glycolipid metabolism and insulin secretion by β-cells through pathways such as mTORC1 and glutamate dehydrogenase (GDH) [74,75]. Leucine, as a core member, can be metabolized into α-ketoisocaproic acid (α-KIC), which directly inhibits the K_ATP_ channels of β cells and collaborates with glucose to promote insulin release. In addition, the short-term activation of mTORC1 by leucine downregulates the inhibitory α_2_A-adrenergic receptor (α_2_AAR) on the β-cell membrane, indirectly enhancing insulin secretion, while rapamycin can block this process [76,77,78]. Moreover, leucine can allosterically activate GDH and upregulate ATP synthase β subunit (ATPβ) and glucokinase (GK) over the long term, thereby enhancing the metabolic capacity of β-cells [76,77,78]. However, when plasma BCAAs levels rise, the chronic activation of mTORC1 can lead to excessive phosphorylation of insulin receptor substrate 1 (IRS1), impaired insulin signaling, and an increased risk of insulin resistance and T2D [79].

Incretins from the intestine provide another key regulatory layer for liver–pancreatic communication. Glucagon-like peptide-1 (GLP-1) and glucose-dependent insulinotropic polypeptide (GIP) can enhance insulin secretion stimulated by glucose, inhibit excessive hepatic glucose production, and improve hepatic insulin sensitivity [80]. They integrate intestinal nutritional stimulation, liver metabolic status and pancreatic islet response to form a three-way communication network of gut–liver–pancreas. Due to their multiple metabolic regulatory effects in MASLD and T2D, GLP-1 and GIP have become highly attractive therapeutic targets, and their receptor agonists have achieved remarkable clinical results.

Bile acids are a class of endocrine-active signaling molecules that regulate energy metabolism by connecting the liver, intestine and pancreas [81,82]. After bile acids activate the nuclear farnesoid X receptor (FXR) in the liver, they can inhibit lipid production and promote fatty acid oxidation, thereby improving the metabolic state of the liver. When the membrane Takeda G protein-coupled receptor 5 (TGR5) expressed in pancreatic β cells is activated, it can directly enhance insulin secretion [83,84]. Furthermore, the activation of TGR5 by bile acids in the intestine promotes the release of GLP-1, thereby indirectly improving glucose homeostasis [82,84]. Metabolic diseases such as MASLD and obesity can lead to changes in bile acid composition, abnormal activation of TGR5 or FXR, and reduced secretion of GLP-1, ultimately damaging liver–pancreatic communication and exacerbating metabolic imbalance [85,86].

### 2.4. Adipokines

Adipokines, as important metabolic messengers, link adipose tissue to the liver–pancreas axis, in which they affect lipid processing, insulin sensitivity and β-cell function. Among these factors, adiponectin and leptin have the most distinct characteristics. In hepatocytes, adiponectin mainly transmits signals through its receptors adiponectin receptor 1 and 2 (AdipoR1 and AdipoR2), activating the downstream AMPK and PPARα pathways, thereby enhancing fatty acid oxidation, reducing lipid accumulation and improving glucose metabolism [87,88]. Meanwhile, the activation of AdipoR triggers receptor-associated ceramidase activity, promoting deleterious ceramide degradation, which is a key mechanism by which adiponectin improves systemic insulin resistance and supports cell survival [89]. In the pancreas, adiponectin protects β-cell through direct and indirect mechanisms: the direct mechanism is to activate the AdipoR-APPL1-Akt/ERK pathway to enhance insulin biosynthesis and inhibit β-cell apoptosis [90], whereas hepatic leptin signaling via JAK/STAT-SOCS3 and PI3K pathways regulates insulin sensitivity, while direct leptin signaling in pancreatic β-cell inhibits insulin secretion, collectively contributing to the prevention of β-cell overload in states of energy surplus [91,92,93].

Beyond adiponectin and leptin, multiple adipokines have been shown to modulate the liver–pancreas axis. Resistin increases glucose production and promotes hepatic insulin resistance [94], thereby indirectly impairing pancreatic β-cell function. Retinol-binding protein 4 (RBP4), derived from both adipose tissue and the liver, promotes hepatic gluconeogenesis and insulin resistance while impairing compensatory β-cell function [95]. Apelin restores glucose tolerance and increases glucose utilization in the short term [96]. However, its levels are often increased in obesity and insulin resistance, suggesting it may act as a compensatory factor, and prolonged elevation could lead to reduced responsiveness to apelin [97]. Omentin, a visceral adipose tissue-derived adipokine, has been shown to act as a protective adipokine in the liver–pancreas axis by improving insulin sensitivity and glucose homeostasis, suppressing hepatic steatosis via AMPKα/mTOR-mediated autophagy, and exerting anti-inflammatory effects that collectively support coordinated metabolic regulation between the liver and pancreas [98,99].

### 2.5. MicroRNA and Extracellular Vesicular Communication

In addition to classic hormones and metabolites, microRNAs (miRNAs) and extracellular vesicles (EVs) have become important mediators of the liver–pancreas axis. More and more evidence indicates that miRNAs and EV-encapsulated miRNAs act as signal vectors to transmit metabolic stress between organs. Mechanistic studies in cellular systems and animal models have shown that hepatocytes under steatotic or insulin-resistant conditions secrete EVs rich in specific miRNAs, such as members of the miR-126 and miR-29 families, which are taken up by pancreatic β-cells. These miRNA-containing vesicles activate inflammatory and stress response pathways including NF-κB and JNK, ultimately impairing β-cell function and reducing insulin secretion capacity [100,101,102,103]. Conversely, pancreatic β-cells have also been shown, primarily in in vitro and rodent studies, to release EVs carrying miRNAs such as miR-29a [101]. These vesicles feed back to the liver and inhibit the IRS-1/PI3K/Akt insulin signaling pathway, thereby exacerbating the metabolic imbalance of the liver–pancreas axis. However, it should be noted that most of the current evidence is derived from preclinical and mechanistic studies, and direct validation in human subjects remains limited. Further studies are required to establish the physiological and clinical relevance of EV-mediated miRNA signaling in the liver–pancreas axis.

**Figure 1:**
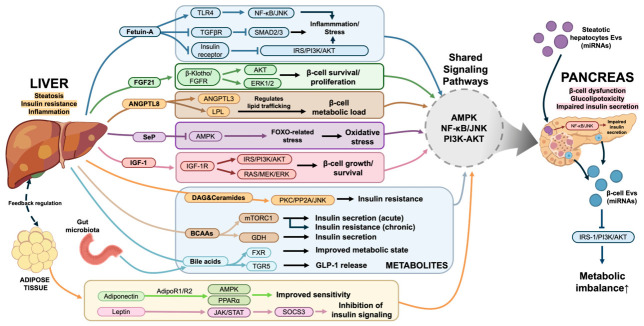
Integrated signaling network of hepatokines, metabolites, adipokines, and EVs in the liver–pancreas axis. The liver and pancreas communicate through hepatokines, metabolites, adipokines, and EVs. Dysregulation of these pathways contributes to insulin resistance, β-cell dysfunction, and metabolic disease progression. Arrows indicate activation, and blunt-ended lines indicate inhibition. TLR4, Toll-like receptor 4; TGFβR, Transforming growth factor β receptor; IRS, Insulin receptor substrate; PI3K, Phosphatidylinositol 3-kinase; AKT, Protein kinase B; FGF21, Fibroblast growth factor 21; FGFR, Fibroblast growth factor receptor; LPL, Lipoprotein lipase; SeP, Selenoprotein P; AMPK, Adenosine monophosphate-activated protein kinase; FOXO, Forkhead box O; IGF-1, Insulin-like growth factor 1; IGF-1R, Insulin-like growth factor 1 receptor; DAG, Diacylglycerol; BCAAs, Branched-chain amino acids; GDH, Glutamate dehydrogenase; FXR, Farnesoid X receptor; TGR5, Takeda G protein-coupled receptor 5; GLP-1, Glucagon-like peptide-1; PPARα, Peroxisome proliferator-activated receptor α; SOCS3, Cytokine signal suppressor 3; EVs, Extracellular vesicles; miRNAs, MicroRNAs. The illustrations are created using BioRender software (https://BioRender.com/3yqw77b (accessed 6 April 2026)).

Beyond their individual roles, hepatokines, metabolites, adipokines, and EVs form an integrated and interactive signaling network within the liver–pancreas axis (Figure 1). These mediators not only exert direct effects on target organs but also influence each other’s production and activity [104]. These mediators can regulate each other’s secretion and activity, and often converge on shared downstream pathways such as AMPK, PI3K-AKT, and NF-κB/JNK signaling, thereby coordinately modulating insulin sensitivity, inflammation, and β-cell function [105]. For example, the hepatokine FGF21 has been shown to stimulate adiponectin expression, demonstrating direct functional crosstalk between liver-derived and adipose-derived factors [104]. Moreover, the liver and adipose tissue engage in bidirectional communication, further supporting the concept of an integrated multi-organ regulatory axis [106].

## 3. Pathophysiology of the Axis in Diabetes

### 3.1. Hepatic Insulin Resistance and Impaired Glucose Homeostasis

The liver is at the core of maintaining the balance of blood glucose throughout the body. When hepatocytes fail to correctly sense insulin signals, the metabolic regulation of the liver–pancreas axis is first disrupted at the liver level and further exerts a chain reaction on the islets. As a result, hepatic insulin resistance has become one of the earliest and most crucial defects in the pathogenesis of T2D.

Mechanistically, hepatic insulin resistance is driven by multiple cellular stress pathways that disrupt insulin signaling. Chronic inflammatory activation mediated by JNK and IKKβ/NF-κB, together with abnormal endoplasmic reticulum–mitochondrial communication, contributes to this impairment [107,108,109]. These molecular mechanisms, when superimposed, cause hepatocytes to lose their normal response to insulin, thereby driving disorders in glucose metabolism. It is worth noting that hepatic insulin resistance is not only a local abnormality but also expands its pathological effects by affecting the secretion of hepatokines. Insulin-resistant hepatocytes can alter secretion of hepatokines such as fetuin-A, ANGPTL8 and FGF21, which in turn regulate insulin secretion and adaptive growth of β cells [110,111].

### 3.2. Hyperglucagonemia and Glucagon Resistance

Persistent hyperglucagonemia usually results from α-cell dysfunction and the failure of insulin, amylin and other local regulatory mechanisms within the islets [112,113]. While glucagon levels keep rising, the liver’s response to its signals weakens, which is known as hepatic glucagon resistance, characterized by a decrease in the reactivity of the cAMP-PKA pathway. Due to impaired signal transduction, glucagon weakens the catabolism of amino acids, causing amino acids to accumulate in the circulation. Elevated amino acids further promote the proliferation of α cells and glucagon secretion, thereby forming the “liver–α-cell axis” [114,115]. Eventually, a positive feedback loop is formed, and hyperglucagonemia will persist. And it further stimulates the liver to produce excessive glucose, leading to hyperglycemia.

### 3.3. MASLD and Its Impact on β-Cell Function

MASLD also plays a key role in the liver–pancreas axis of diabetes. Clinically, the severity of hepatic steatosis is associated with impaired insulin secretion and decreased compensatory capacity of β cells [116,117].

Hepatic lipid accumulation reshapes the liver–pancreas axis by altering the circulating profiles of hepatokines, lipids, and amino acids, ultimately affecting pancreatic β-cell function. In MASLD, the levels of pathogenic hepatokines such as fetuin-A and SeP increase, which promotes systemic insulin resistance and directly damage β-cell function through inflammatory signaling, disruption of insulin receptor pathways, redox imbalance, and inhibition of β-cell maturation and survival [56,118]. Conversely, FGF21 exerts a protective effect on β-cells by enhancing insulin biosynthesis and preventing apoptosis; however, under chronic metabolic stress, its signaling is often weakened due to the downregulation of the β-Klotho/FGFR complex [119,120]. In addition to hepatokines, MASLD also alters the liver–pancreas axis through dysregulation of metabolites and bile acids, further exacerbating β-cell dysfunction. The accumulation of lipids (such as DAGs and ceramides) can trigger hepatic insulin resistance by impairing IR-Akt signaling and activating stress pathways [121], thereby increasing systemic glycolipid toxicity and promoting β-cell apoptosis. In MASLD, dysregulation of bile acid signaling leads to impairment of the liver–gut–pancreas communication mediated by FXR and TGR5, thereby resulting in insulin secretion defects and metabolic homeostasis imbalance [82]. In addition, hepatic steatosis promotes the formation of a pro-inflammatory environment because activated Kupffer cells and infiltrating macrophages release cytokines such as tumor necrosis factor-α (TNF-α), interleukin-6 (IL-6), and interleukin-1 β (IL-1β). These molecules enter the circulation and reach the pancreas, further damaging insulin secretion while promoting inflammation [122,123].

### 3.4. Defects in Hepatic Insulin Clearance

Hepatic insulin clearance is a key process for maintaining physiological circulating insulin levels. Under normal conditions, the liver eliminates approximately 50–80% of the secreted insulin through receptor-mediated endocytosis, followed by lysosomal degradation [124,125]. However, in a state of insulin resistance, this clearance mechanism will be impaired due to the reduced internalization of insulin receptors and changes in their degradation capacity. The resulting hyperinsulinemia may initially compensate for insulin resistance, but it will eventually lead to β-cell exhaustion and exacerbate peripheral insulin resistance [124,126]. Newly emerged evidence further indicates that lipid remodeling that occurs during diabetes [127], such as changes in the ratio of phosphatidylcholine (PC) to phosphatidylethanolamine (PE) caused by choline/ethanolamine phosphotransferase 1 (CEPT1) deficiency, can alter membrane fluidity and insulin receptor transport, thereby affecting the efficiency of insulin clearance in the liver [128,129,130,131,132,133,134].

### 3.5. Intestinal Dysbiosis and the Gut–Liver–Pancreas Axis

Emerging evidence suggests that intestinal dysbiosis is a critical contributor to the dysregulation of the liver–pancreas axis in metabolic diseases. Small intestinal bacterial overgrowth (SIBO) and compositional alterations of the gut microbiota are highly prevalent in MASLD and correlate with disease severity [135]. Mechanistically, disruption of gut barrier integrity promotes the translocation of bacterial components such as lipopolysaccharide (LPS) into the portal circulation, triggering hepatic inflammation through TLR4 signaling [136,137]. This inflammatory milieu exacerbates hepatic insulin resistance and alters hepatokine secretion, thereby indirectly impairing pancreatic β-cell function [136,138]. In addition, gut microbiota modulates bile acid composition and signaling pathways such as FXR and TGR5, which are known to regulate both hepatic glucose metabolism and insulin secretion [82]. Therefore, gut dysbiosis acts as an upstream driver linking intestinal dysfunction to coordinated hepatic and pancreatic impairment.

## 4. Clinical Implications Across Different Diabetes Types

### 4.1. Type 2 Diabetes: Insulin Resistance, Fatty Liver, and Glucagon Excess

T2D is characterized by early and severe disorder of the liver–pancreas axis, in which the liver becomes the core driver of both fasting and postprandial hyperglycemia. This dysfunction reflects the combined effect of insulin resistance in the liver, lipid overload, β-cell damage and inappropriate glucagon signaling, and these factors reinforce each other on this axis.

Hepatic insulin resistance is a major initial defect in the pathogenesis of T2D. Under normal circumstances, insulin inhibits gluconeogenesis in the liver and promotes glycogen synthesis. However, in T2D, the IR/insulin receptor substrate (IRS)/AKT signaling cascade is impaired, leading to insufficient phosphorylation of FOXO1, which maintains its transcriptional activity in the nucleus and continuously drives the expression of gluconeogenic genes such as PEPCK and G6Pase [20]. Meanwhile, the weakened inhibitory effect on glycogen synthase kinase 3β (GSK3β) will reduce glycogen synthesis, while the impaired activity of glucokinase (GCK) will disrupt the conversion of glucose to glucose-6-phosphate (G6P), indirectly promoting gluconeogenesis [20]. To make matters worse, the glucagon pathway is often overactivated, and upon binding to its GPCR, glucagon increases intracellular cAMP levels and activates the PKA/CREB pathway, thereby further enhancing the transcriptional process of gluconeogenesis [139]. Muscle insulin resistance reduces glucose uptake and glycogen storage, indirectly increasing the liver’s dependence on non-carbohydrate precursors [20,140]. Therefore, in the case of hepatic insulin resistance, the ability of insulin to inhibit glucongenesis is lost, and multiple interacting mechanisms jointly maintain the chronic increase in hepatic glucose output.

In addition, peripheral insulin resistance, particularly in adipose tissue, attenuates insulin-mediated suppression of lipolysis, leading to overproduction of free fatty acids (FFAs). Enhanced fat breakdown will transport a large amount of FFAs to the liver and lead to lipid accumulation within hepatocytes [141]. Excessive circulating free fatty acids enter β-cells through transporters such as CD36, leading to lipid accumulation within the cells and disrupting the synthesis and secretion of insulin. Lipotoxicity can simultaneously induce ER stress, oxidative stress and β-cell apoptosis, reducing the number of functional β-cells [141,142,143]. Steatosis hepatocytes also release inflammatory cytokines and EVs that can impair the insulin signaling and insulin secretion of β cells [102,144].

Furthermore, the dysregulation of glucagon is involved in the dysregulation of the liver–pancreas axis. In T2D, α-cells secrete excessive glucagon, and the liver’s reactivity to glucagon is enhanced, which promotes liver glucose production even in fed state [145,146,147]. This dual dysregulation can lead to hyperglycemia and further increase the burden on β cells.

### 4.2. Type 1 Diabetes: Hepatic Insulin Deficiency and Altered Glucagon Response

In T1D, the complete absence of endogenous insulin secretion fundamentally disrupts the physiological connection between the pancreas and the liver. Under normal circumstances, insulin delivered through the portal vein has a significant inhibitory effect on hepatic glucose production. However, when this direct signal is absent, hepatic glucose metabolism will be disrupted.

One of the main consequences of insulin deficiency is the continuous increase in hepatic glucose output. In hepatocytes lacking insulin signaling, gluconeogenesis enzymes (such as PEPCK and G6Pase) are inappropriately and continuously upregulated, while impaired phosphorylation of glycogen synthesis-related enzymes (including GSK3β and glycogen synthase) significantly reduces glycogen production [32,148]. Further animal studies have shown that even in the presence of systemic hyperglycemia, insulin-deficient livers continue to drive excessive gluconeogenesis, which largely leads to glucose fluctuations in T1D patients [148]. Reduced liver glycogen reserves further impair the ability to maintain stable glucose levels during fasting or exercise, intensifying fluctuations in glucose levels [32].

Glucagon dynamic disorder is another significant feature of liver–pancreas communication disorder in T1D. Due to α-cell dysfunction, the body’s glucagon response to hypoglycemia is significantly weakened. Therefore, T1D patients rely more on anti-regulatory hormones such as adrenaline and cortisol, which act more slowly and are less effective. This makes hypoglycemic episodes last longer and significantly increases the risk of serious complications, including hypoglycemic coma and seizures [149,150]. On the contrary, during or after hyperglycemia, many patients will experience persistent and abnormal hyperglucaginemia. In patients with T1D, due to the lack of inhibitory signals produced by β-cells, excessive glucagon secretion persists, thereby increasing liver glucose production and exacerbating postprandial hyperglycemia. Chronic postprandial hyperglucagon syndrome may also lead to insulin resistance in the liver, intensifying the vicious cycle of hyperglycemia and insulin resistance [151,152].

### 4.3. Gestational Diabetes: Hepatic Adaptations in Pregnancy and Insulin Secretion

During normal pregnancy, the maternal liver undergoes profound metabolic reprogramming to meet the increasing energy demands of the growing fetus. Placental hormones and cytokines are the main regulatory factors for reshaping metabolic pathways in adipose tissue, skeletal muscle and liver. These signals can reduce the utilization rate of glucose in the mother’s periphery, enhance lipolysis, and stimulate hepatic gluconeogenesis, jointly leading to elevated levels of hyperglycemia and FFAs in the mother, thereby ensuring the delivery of adequate nutrition to the fetus [153,154]. This process is accompanied by progressive insulin resistance, which is most obvious in the middle and late stages of pregnancy. By the late stage of pregnancy, the mother’s insulin sensitivity drops to 50–70% of the pre-pregnancy level, and it is necessary to compensate by increasing insulin secretion to maintain normal glucose levels [155,156]. To maintain glucose homeostasis in these circumstances, maternal β-cells proliferate and increase insulin secretion, which is part of compensatory adaptation [157]. When this adaptive response is insufficient, maternal hyperglycemia or GDM may occur [158].

Metabolic regulation related to pregnancy depends on a complex network mediated by hormones, cytokines, insulin-like growth factor binding proteins (IGFBPs), and lipid signaling among the placenta, liver, and pancreas. This network can be disrupted in GDM. Placental hormones, such as human placental prolactin (hPL), placental growth hormone (PGH), leptin and resistin, play the role of core regulatory factors. In GDM, excessive secretion of PGH, leptin and resistin can lead to extensive metabolic dysfunction. During pregnancy, PGH gradually increases as the main form of GH in the maternal circulation, and it has been proposed to be involved in the formation of insulin resistance during pregnancy [159]. Resistin can induce the expression of cytokine signal suppressor 3 (SOCS3), inhibit the phosphorylation of AKT, reduce insulin release, and lead to insufficient compensation of β-cells [160]. Elevated leptin levels may further aggravate liver insulin resistance by enhancing the gluconeogenic process [161]. In addition, the placenta of women with GDM secretes more TNF-α and IL-6. These substances can damage insulin secretion in the pancreas, enhance gluconeogenesis and lipolysis in the liver, and form a cycle where inflammation and metabolic dysfunction reinforce each other [162]. Clinical and histological studies suggest that GDM is not merely a disorder of maternal glucose metabolism, but is accompanied by pronounced placental inflammation and endocrine remodeling. In GDM placentas, components of the IL-1β and TLR signaling pathways, including IL1R1, IL1RAP, TLR4, CD14, and NF-κB, are upregulated, indicating heightened immune activation [163]. IL-1β- and TLR-driven inflammatory signaling promotes the local production of pro-inflammatory cytokines such as IL-6 and TNF-α and establishes autocrine feedback loops that sustain sterile inflammation [163]. These placental inflammatory adaptations may indirectly influence hepatic insulin sensitivity and β-cell function by altering the placental hormonal and cytokine milieu [163], highlighting the potential importance of assessing placental hormone levels, inflammatory markers, and maternal β-cell function for early risk identification.

Long-term follow-up studies, most notably the Study of Women, Infant Feeding, and Type 2 Diabetes After GDM Pregnancy (SWIFT), have consistently demonstrated that women with a history of GDM carry a substantially elevated risk of developing T2D later in life [164]. It is worth noting that the SWIFT study also observed that during the transition to T2D, postpartum women often experienced significant increases in serum TAGs, as well as insufficient synthesis of phospholipids and sphingolipids. These metabolic changes were already evident before the onset of T2D [165], highlighting the existence of functional imbalances among multiple metabolic organs during the postpartum period of GDM. Before obvious postpartum metabolic deterioration occurs, many women have already shown persistent liver insulin resistance and impaired β-cell compensatory function [166,167]. Circulating hepatokines, such as FGF21 and fetuin-A, have become potential early biomarkers of this long-term risk. Elevated levels of maternal FGF21 may indicate a compensatory response to metabolic stress, while elevated levels of fetuin-A promote insulin resistance by inhibiting insulin receptor signaling [168,169].

### 4.4. Monogenic and Rare Forms: Genetic Disruption of Hepatic–Pancreatic Regulation

Monogenic diabetes and rare genetic diseases offer a unique perspective on the genetic integrity required for the normal synergy of the liver and pancreas. Mutations that affect glucose perception, transcriptional regulation or lipid processing selectively disrupt metabolic communication between the liver and pancreas, thereby leading to early-onset or atypical diabetic phenotypes.

The most common monogenic forms, MODY2 (maturity-onset diabetes of the young), MODY3 and MODY1, are respectively caused by pathogenic variations of the GCK, hepatocyte nuclear factor 1-alpha (HNF1A) and hepatocyte nuclear factor 4-alpha (HNF4A) genes. These genes regulate both pancreatic β-cell function and hepatic metabolic pathways. In MODY2 or permanent neonatal diabetes mellitus (PNDM), loss-of-function mutations in the GCK gene can reduce enzyme activity or glucose affinity and increase the glucose threshold for insulin secretion. Insufficient insulin secretion can weaken liver glycogen synthesis and enhance gluconeogenesis, delay the clearance of postprandial glucose, and lead to mild chronic hyperglycemia, and may further cause liver steatosis and progressive β-cell stress [170,171]. HNF1A and HNF4A encode transcription factors that are co-expressed in the liver and pancreas. Their destruction will alter aspects involving insulin synthesis, glucose transport and lipid metabolism. In the liver, the HNF1A mutation reduces the expression of glucose-6-phosphate transporter (G6PT), disrupts gluconeogenesis regulation, and thereby leads to fasting hyperglycemia [172,173]. These mutations also alter the process by which the liver secretes signaling molecules such as C-reactive protein (CRP), indirectly regulating the sensitivity of β cells [172,173]. In the pancreas, mutations in HNF1A or HNF4A can impair insulin secretion and survival of β cells, resulting in the progressive insulin reduction feature commonly found in MODY1 that occurs in adolescents [174]. Patient-derived models have shown impaired β-cell differentiation, reduced glucose-stimulated insulin secretion (GSIS), and disordered islet structure, highlighting the combined impact of these transcriptional defects on the liver and pancreas [174,175]. Several other monogenic defects mainly affect β-cell differentiation, KATP channel function or ER stress homeostasis, such as mutations in PDX1, KCNJ11/ABCC8, INS and WFS1 genes [176,177,178,179]. Although these genes mainly function within β cells, the resulting insulin deficiency can subsequently affect insulin signaling in the liver.

In contrast, a unique type of rare diabetes-related variant mainly originates from the liver, indicating that liver metabolic damage may lead to endocrine failure of the pancreas. Mutations in lipid processing genes (including CEPT1, MTTP and PNPLA3) can disrupt phospholipid remodeling, the assembly/secretion of very low-density lipoprotein (VLDL), and lipid droplet turnover [132,180,181]. Impaired VLDL output or alterations in phospholipid composition can promote liver lipid retention, ER stress and insulin resistance. These liver defects can enhance gluconeogenic output, increase systemic lipid flow, impose chronic metabolic stress on β cells, and accelerate their functional disorders [132,180,181]. Hereditary lipodystrophy caused by mutations in the LMNA (familial partial lipodystrophy) or AGPAT2 (congenital systemic lipodystrophy) genes further indicates that defects in adipose tissue development can lead to the ectopic accumulation of excess lipids in organs such as the liver and pancreas [182,183]. Severe hepatic steatosis, hypertriglyceridemia and hepatic insulin resistance jointly increase the workload of β cells, while pancreatic fat infiltration directly leads to impaired insulin secretion [182,183].

### 4.5. MASLD as a Predictor of Diabetes Onset

More and more clinical evidence indicates that MASLD is not only a manifestation of metabolic syndrome in the liver but also a strong predictor of future T2D [184,185]. Compared with people without fatty liver, those with hepatic steatosis have a significantly higher risk of progressing to diabetes, highlighting its role as an early metabolic warning signal [185,186].

Clinically, MASLD is regarded as an early indicator of diabetes risk, providing an opportunity for proactive screening and intervention [186]. Incorporating liver fat assessment into routine metabolic evaluations may help identify high-risk groups earlier and guide timely preventive strategies. From a mechanism perspective, excessive accumulation of lipids in the liver can damage the insulin signaling pathway, leading to insulin resistance in the liver and increasing the metabolic pressure on pancreatic β-cells. Newly emerged studies also indicate that FGF21 resistance and mild hepatogenic inflammation occur early in prediabetes, further accelerating systemic abnormal glucose regulation [187].

## 5. Therapeutic Perspectives

### 5.1. Lifestyle Interventions

Lifestyle changes are the cornerstone for restoring normal metabolism along the liver–pancreas axis. Dietary intervention, especially calorie restriction, can significantly reduce hepatic steatosis and improve insulin clearance in the liver [188]. In clinical research, even simple calorie restriction (without exercise) can significantly reduce TAG content and decrease insulin demand, thereby alleviating β-cell stress [189]. Low-carbohydrate diet/ketogenic diet can rapidly reduce hepatic fat in the short term, but long-term use may cause lipid profile disorders, liver fat accumulation, insulin resistance, and even aggravate MASLD [190,191]. Unlike the extremely low-carbohydrate ketogenic diet, the Mediterranean diet is a dietary pattern that mainly consists of plant-based foods while minimizing the intake of refined carbohydrates. A key feature of this diet is the emphasis on monounsaturated and polyunsaturated fatty acids, which can inhibit de novo lipogenesis in the liver and promote β-oxidation in mitochondria, thereby significantly reducing the accumulation of TAG, improving insulin sensitivity, and thus lowering lipid toxicity to β-cells and reducing the workload of β-cells [192]. Another effective method is time-restricted feeding, which keeps nutrient intake consistent with the circadian metabolic rhythm. This dietary pattern enhances the thermogenesis of brown adipose tissue (BAT), thereby increasing energy expenditure and reducing lipids entering the liver [193]. At the same time, it can also reduce the accumulation of ectopic fat and alleviate the inflammatory damage caused by lipid ectopic deposition to organs such as the liver, cardiovascular system and pancreas [193].

Exercise is another effective means of intervention. Regular aerobic training and resistance exercise can enhance fatty acid oxidation in liver mitochondria, thereby reducing liver TAG deposition and inhibiting the expression of ER stress-related genes such as Atf4 and Chop, maintaining ER homeostasis [194,195]. Exercise can also promote the uptake of glucose by peripheral tissues, enhance insulin sensitivity throughout the body, and reduce the burden on β cells. Further mechanism research indicates that exercise can activate the Sirt1/PGC-1α pathway, enhance the biosynthesis of liver mitochondria, counteract lipid toxicity and ER stress, and jointly form a more benign liver–pancreas metabolic cycle [195]. In addition, exercise can also increase the expression of the Fgf21 gene. As a hepatokines, FGF21 can induce enhanced mitochondrial function and fatty acid oxidation, associating the improvement of liver metabolism with the regulation of systemic metabolism [194,195].

Clinical trials have shown that a weight loss of 5–10% can significantly reduce transaminase levels and hepatic fat content and enhance insulin secretion capacity [196]. Bariatric surgery can also bring multi-dimensional benefits, as it not only significantly improves the structure and function of the liver, but also enhances β-cell function and insulin resistance [197,198]. The adiponectin/leptin ratio (Adpn/Lep) increased after the operation, and this ratio was negatively correlated with hepatic inflammation and insulin resistance, further indicating that bariatric surgery helps restore metabolic communication between the liver and the pancreas [197].

### 5.2. Current Pharmacological Interventions with Dual Effects

More and more evidence indicates that drugs targeting metabolic diseases have an effect on both the liver and pancreas, thereby regulating the liver–pancreas axis as a whole.

Metformin is widely used as a first-line drug for the treatment of T2D. Metformin synergistically reduces blood glucose through two pathways: inhibiting endogenous glucose production and enhancing the uptake of glucose by peripheral muscle and adipose tissues. In addition, metformin can also inhibit liver fat synthesis and promote fat decomposition and oxidation, improving the common dyslipidemia in patients with T2D [199]. These effects reduce lipid accumulation in the liver, repair insulin signaling pathways and alleviate systemic inflammatory stress signals, thereby effectively improving liver insulin sensitivity. Although metformin does not directly promote insulin secretion, it indirectly protects the integrity of β cells by reducing systemic glucose and lipid loads, alleviating oxidative stress and endoplasmic reticulum stress, and alleviating chronic inflammation [200]. By improving the metabolic state of the whole body, metformin helps the survival of β cells and maintains the insulin secretion capacity stimulated by glucose [200].

SGLT2 inhibitors (SGLT2i) reshape this axis through a unique non-insulin-dependent mechanism. This drug reduces plasma glucose by increasing glucose excretion in the kidneys without the need for insulin secretion, thereby reducing the burden on β cells and alleviating glucose toxicity stress. Over time, this remission effect enhances the adaptability and functional stability of β cells [201]. Meanwhile, SGLT2i can also trigger systemic metabolic adaptations, improving insulin sensitivity in the liver and reducing steatosis injury by increasing ketone body utilization, enhancing FGF21 signaling, and promoting lipid oxidation in the liver [202]. These secondary liver responses create a more favorable hormonal and metabolic environment for the maintenance of β cells, which is conducive to the recovery of the liver–pancreas communication circuit.

GLP-1 receptor agonists (GLP-1RAs) can regulate liver and pancreas physiology at the same time. The pancreas is one of the core target organs of GLP-1RA. In β cells, the activation of GLP-1R enhances glucose-stimulated insulin secretion through the cAMP-PKA and PI3K-AKT pathways, and promotes the proliferation and survival of β cells [203]. The liver is not a high-expression organ of GLP-1R, but GLP-1RA improves liver metabolic disorders by increasing the cAMP level in liver cells, AMPK, inhibiting hepatic fat production and accelerating lipolysis [204]. In addition, although GLP-1RA does not directly act on adipose tissue, its insulin-promoting effect can reduce the transport of FFA to the liver by inhibiting fat lipolysis and enhancing fat uptake of substrates [203]. It can also suppress appetite and reduce total calorie intake through the central nervous system to decrease visceral fat [203]. These two pathways jointly improve adipose–liver crosstalk and, together with the direct activation of the liver AMPK pathway, constitute the liver-protective effect of GLP-1RA.

Another treatment strategy involves multi-agonist insulin gonadotropin mimics, particularly GLP-1/glucagon dual agonists and GLP-1/GIP/glucagon triple agonists. Polyagonists inhibit hepatic lipid synthesis and promote lipid oxidation by binding to glucagon receptors, significantly reducing hepatic fat content. They can also maintain β-cell function through GLP-1R signaling [205,206]. By reducing liver steatosis, enhancing mitochondrial efficiency and lowering systemic insulin requirements, a metabolic environment conducive to the long-term survival of β cells has been jointly created [205,206]. Recently, Phase 2 clinical trials of multiple agonists such as retatrutide have shown that they can significantly reduce liver lipid content in a dose-dependent manner. A considerable proportion of the subjects achieved normalization of liver lipid, accompanied by obvious weight loss, improved blood glucose control, and reduction in visceral fat [207].

### 5.3. Emerging Therapies Targeting the Liver–Pancreas Axis

Compared with the aforementioned antidiabetic drugs, emerging metabolic therapies are increasingly focusing on restoring the coordinated information exchange between the liver and the pancreas, aiming to simultaneously correct hepatic lipid metabolism, systemic insulin dynamics, and the functional integrity of β-cell.

Due to the short half-life of FGF21, FGF21 analogs that extend the circulating half-life while retaining biological activity are among the most advanced therapeutic drugs in this category. FGF21 can inhibit hepatic lipid synthesis, promote the reduction of hepatic TAG and plasma TAG levels, alleviate hepatic steatosis, and improve hepatic insulin sensitivity, thereby indirectly reducing the lipotoxic stress of pancreatic β cells [208]. Meanwhile, its direct protective effect on the islets can further improve the disorder of glucose and lipid metabolism in the liver by increasing insulin secretion and reducing glucagon [208]. Animal studies have also shown that the FGF21-adiponectin-ceramide axis serves as a potential mediator for protective signal transduction from the liver to β cells [209]. FGF21 can significantly promote the secretion of adiponectin in adipose tissue and simultaneously reduce the level of ceramide in obese mice, thereby achieving the purpose of reducing lipotoxic mediators and alleviating the pressure on β cells [209]. Furthermore, studies on long-acting analogs and dual agonists (e.g., GLP-1/FGF21 fusion peptides) have further highlighted the therapeutic potential targeting liver–pancreas metabolic integration [210]. In addition to FGF21, hepatokines such as fetuin-A and ANGPTL8 have also become key messengers linking liver lipid stress with β-cell inflammation and secretory dysfunction, which makes it possible for liver factor antagonism or replacement therapy to restore axial homeostasis.

The exosome-based strategy offers a novel therapeutic approach by regulating inflammation, lipid metabolism, and key inter-organ signaling pathways. The core mechanism relies on the activation of the FGF21–adiponectin axis and the delivery of functional miRNAs. These combined effects can improve lipid accumulation and inflammatory states in the liver, while supporting the survival and function of pancreatic β cells [211,212]. These advancements highlight the potential of exosome-mediated therapies in treating metabolic disorders involving liver–pancreatic communication disorders. Although still preclinical, these methods point toward a future in which fine-scale manipulation of liver–pancreas communication could yield personalized therapies.

## 6. Future Directions

### 6.1. Omics Approaches in Liver–Pancreas Axis Research

The latest advancements in multi-omics techniques offer deeper insights into the biological mechanisms behind the liver–pancreas axis. The integrated multi-omics approach, including metabolomics, proteomics, transcriptomics and lipidomics, enables researchers to explore the dynamic and multi-level interactions between the liver and pancreas at the systemic level. Throughout the entire disease spectrum from MASLD to T2D, multi-omics approaches can reveal key molecular signals, such as specific lipid mediators, amino acid-derived metabolites, and hepatokines, and clarify how they have cross-organ effects on pancreatic islet function and systemic insulin sensitivity [213]. In addition, these methods also help identify potential disease drivers, thereby laying the foundation for classification and precise intervention. For GDM, multi-omics techniques can identify early biomarkers and predict the risk of postpartum progression to T2D. The data from the SWIFT cohort show that metabolic disorders already existed in the early postpartum period before obvious glucose abnormalities occurred. Women who later developed T2D had elevated levels of BCAAs and hexoses in their circulation, and had unique lipidomic characteristics, manifested as increased TAGs and DAGs, as well as decreased specific phospholipid and sphingolipid species. Importantly, the metabolic features excelled traditional glucose detection indicators in predicting the risk of future T2D [165,214,215,216].

Spatial omics techniques, such as spatial transcriptomics and spatial proteomics, have further deepened our understanding of the structure and function of these organs and tissues. In studies of the liver and pancreas, spatial transcriptomics has revealed functional specialization of distinct cellular subpopulations within specific spatial microenvironments and their roles in metabolic regulation [217,218]. In MASLD patients, spatial omics has clarified the regional enrichment of steatosis, immune cell remodeling, and fibrotic signaling within defined hepatic zones, demonstrating that disease progression is driven by local microenvironmental alterations rather than uniform dysfunction across the entire organ [217]. In T2D, spatial transcriptomic analyses further uncover disruption of the endocrine–exocrine boundary, accumulation of inflammatory cells surrounding islets, and abnormal spatial distribution of hormone-related transcripts, indicating a close association between islet dysfunction and disorganization of tissue architecture [218].

Single-cell omics is also an indispensable tool for analyzing cellular heterogeneity within the liver–pancreas axis. In studies of the liver–pancreas axis, single-cell omics have revealed functional diversification of immune and parenchymal cell populations in both organs [219,220]. In MASLD, single-cell omics have identified disease-driving hepatocyte subpopulations, reprogramming of macrophages and stellate cells, and epigenetically regulated transitions from inflammation to fibrosis, highlighting that disease progression is governed by specific cellular states rather than uniform hepatic dysfunction [219]. In T2D, single-cell omics further uncover selective loss of high-function β-cell subtypes, transcription factor-dependent regulatory defects, and immune dysregulation within the islet microenvironment, providing mechanistic insight into impaired insulin secretion and insulin resistance [220]. However, the omics studies mentioned above have not yet clearly elucidated the mechanisms by which the liver–pancreas axis influences each organ.

### 6.2. Experimental Models for Studying the Liver–Pancreas Axis

Recent advances in experimental models are expected to significantly accelerate research on the liver–pancreas axis. Three-dimensional organoid systems derived from pluripotent or adult stem cells have been increasingly used to simulate the interaction between the liver and pancreas and the pathophysiological processes of diabetes. In the pancreatic field, stem cell-derived β-cell organoids have achieved functional maturation characterized by GSIS and dynamic insulin release kinetics. For instance, human pluripotent stem cells can be differentiated into functional β-like cells, which, upon transplantation into diabetic mouse models, demonstrate the capacity to reverse hyperglycemia [221]. In liver research, patient-derived and chemically induced liver organoids have been successfully employed to recapitulate key pathophysiological features of metabolic liver diseases associated with diabetes. These organoid models faithfully reproduce hallmark disease phenotypes, including hepatic lipid accumulation, immune cell infiltration and pro-inflammatory cytokine upregulation, and early-stage fibrotic remodeling. Importantly, they exhibit pharmacologically relevant responses to metabolic therapeutics, including PPAR agonists, thereby validating their utility in mechanistic disease modeling, preclinical drug screening, and therapeutic development [222].

Building upon these single-organoid platforms, co-culture and integrated multi-organoid systems enable modeling of liver–pancreas interactions in a controlled microenvironment. By integrating liver and pancreatic organoids, as well as incorporating non-parenchymal components, these platforms can recapitulate key interactions between hepatocytes, stromal cells, and pancreatic β cells within a controlled microenvironment. Such co-culture and microphysiological systems have been shown to reproduce coordinated glucose-responsive behavior, including GSIS and hepatic insulin signaling, while also capturing pathological features under diabetic conditions, such as impaired glucose utilization, mitochondrial dysfunction, and steatotic changes [13]. Importantly, these dysfunctions can be partially reversed by antidiabetic agents, highlighting the utility of co-culture organoid systems for studying liver–pancreas crosstalk and evaluating therapeutic responses. Compared with single-organoid models, these integrated systems more faithfully mimic tissue complexity and provide a powerful platform for investigating the mechanisms underlying metabolic diseases [13].

Despite the advantages of in vitro models, *in vivo* cross-organ experimental systems remain indispensable for validating findings within a physiologically integrated context, as they incorporate endocrine, neural, immune, and metabolic inputs that cannot be fully recapitulated in vitro. In particular, *in vivo* gene delivery using viral vectors such as adeno-associated virus (AAV) enables efficient and targeted genetic manipulation at the organ level. For example, delivery of AAV2 encoding the pancreas lineage factor PDX1 to a humanized liver mouse model enabled transduction of hepatocytes and amelioration of hyperglycemia in streptozotocin-induced diabetic mice, demonstrating the feasibility of organ-level gene manipulation in metabolic disease modeling [223]. The integration of these advanced in vitro and *in vivo* models is essential for bridging mechanistic insights into metabolic diseases with clinical translation.

### 6.3. Knowledge Gaps and Opportunities for Integrated Treatment

Although remarkable progress has been made in understanding the liver–pancreas axis, there are still many gaps that hinder the transformation of mechanism research into clinical practice. Although multi-omics and imaging studies have revealed the links between organs and disease subtypes, there is still a lack of relevant research on precise molecular mediators and longitudinal dynamic changes. The molecular pathways of communication between the liver and pancreas, including specific lipid types, hepatokines and inflammatory mediators involved in metabolic stress transmission, have not been fully elucidated. In addition, the main drivers of the liver–pancreas axis may vary at different stages of the disease (prediabetes, MASLD and obvious T2D), but longitudinal studies on time dynamics are still limited. Filling these gaps in knowledge is crucial for the research and development of treatment methods. Furthermore, in current clinical practice, there are no clear standards to distinguish between “liver-driven” and “pancreas-driven” diabetes phenotypes. There are still few comparative studies evaluating how antidiabetic drugs regulate the liver–pancreas axis. The current treatment model continues to focus on the management of a single organ and lacks a coordinated cross-organ treatment strategy.

Future research on the liver–pancreas axis should first focus on the following: (1) Utilizing integrated multi-omics and spatial resolution techniques to clarify the exact molecular mediators of cross-organ communication, including lipid types, hepatokines, and inflammatory signals; (2) Establish longitudinal cohorts to map the stage-specific changes in axis function during the transition from prediabetes and MASLD to obvious T2D; (3) Develop mechanistic models, supported by artificial intelligence-driven predictive frameworks, to explain how pancreatic islets sense liver metabolic stress and to convert it into functional changes; (4) Transform these insights into a clinically actionable framework by creating diagnostic tools that can distinguish between liver-driven and pancreas-driven diabetes phenotypes.

## 7. Conclusions

The liver–pancreas axis achieves bidirectional communication through multi-level signals such as insulin/glucagon, hepatokines (such as FGF21, fetuin-A, SeP), metabolites (BCAAs, bile acids, DAG/ceramide), and exosomes/miRNA. Its imbalance constitutes the core pathological basis of metabolic diseases such as MASLD and T2D, and liver steatosis is an important predictor of the occurrence of diabetes. Clinically, lifestyle interventions, along with existing drugs (such as GLP-1RA and SGLT2i), as well as FGF21 analogs and multi-receptor multi-agonists, have shown the potential to simultaneously repair liver and pancreas functions. Future research should focus on the combination of longitudinal multi-omics and spatial omics and explore biomarkers that can promote the transformation of institutional discoveries towards precise stratification and collaborative intervention, in order to achieve individualized diagnosis and treatment strategies for metabolic diseases based on the liver–pancreas axis.

## Figures and Tables

**Table 1:** Major hepatokines and their effects on pancreatic function in the liver–pancreas axis.

Hepatokine	Primary Receptor/ Signaling Pathway	Effect on β-Cell Function	Overall Metabolic Impact	Pathophysiological Role	Refs
Fetuin-A	TLR4 → NF-κB/JNK; TGFβR-SMAD2/3 inhibition; IR autophosphorylation suppression	Impairs β-cell maturation and function; promotes inflammation; inhibits insulin signaling	Promotes systemic insulin resistance and lipotoxic stress	Pathogenic amplifier linking hepatic steatosis to β-cell dysfunction	[18,42,43,44,45,46,47]
FGF21	β-Klotho/FGFR complex → Akt, ERK1/2; PPARα-PGC-1α axis	Enhances insulin biosynthesis; prevents apoptosis; improves β-cell survival	Promotes fatty acid oxidation; improves hepatic insulin sensitivity	Protective hepatokine; signaling often impaired in chronic metabolic stress	[17,48,49,50,51]
ANGPTL8 (Betatrophin)	Interacts with ANGPTL3; modulates LPL activity; Akt-related metabolic signaling	Indirect modulation via lipid redistribution; no confirmed direct β-cell proliferation	Regulates triglyceride trafficking and lipid flux	Nutrient-responsive lipid regulator; indirect contributor to β-cell metabolic load	[52,53,54,55]
SeP	AMPK inhibition; FOXO activation; redox modulation	Impairs insulin secretion; promotes oxidative stress in β cells	Induces systemic insulin resistance	Causal mediator of hepatic insulin resistance and β-cell dysfunction	[56,57,58,59]
IGF-1	IGF1R → IRS-PI3K-AKT; RAS-MEK-ERK pathways	Promotes β-cell survival, proliferation, and insulin secretion	Regulates systemic insulin sensitivity; interacts with GH axis	Adaptive growth and metabolic regulator; dysregulated under chronic stress	[60,61,62,63,64]

The table summarizes key hepatokines reported to regulate pancreatic β-cell function through endocrine signaling within the liver–pancreas axis. These factors influence β-cell survival, insulin secretion, and systemic metabolic homeostasis through diverse signaling pathways, thereby contributing to the development or progression of metabolic disorders such as MASLD and T2D.

## Data Availability

No new data were created or analyzed in this study.

## Abbreviations

The following abbreviations are used in this manuscript:

MASLD	Metabolic dysfunction-associated steatotic liver disease
MAFLD	Metabolic dysfunction-associated fatty liver disease
NAFLD	Non-alcoholic fatty liver disease
T2D	Type 2 diabetes
FGF21	Fibroblast growth factor 21
T1D	Type 1 diabetes
GDM	Gestational diabetes mellitus
IR	Insulin receptor
FOXO1	Forkhead box O1
PEPCK	Phosphoenolpyruvate carboxykinase
G6pase	Glucose-6-phosphatase
GPCR	G protein-coupled receptor
AC	Adenylate cyclase
cAMP	Cyclic adenosine monophosphate
PKA	Protein kinase A
CREB	Cyclic amp-responsive element-binding protein
CRTC2	Creb-regulated transcription coactivator 2
PGC-1α	Peroxisome proliferator-activated receptor γ co-activator 1α
GCGR	Glucagon receptor
TLR4	Toll-like receptor 4
FGFR	Fibroblast growth factor receptor
LPL	Lipoprotein lipase
TAG	Triglyceride
SeP	Selenoprotein P
AMPK	Adenosine monophosphate-activated protein kinase
IGF-1	Insulin-like growth factor 1
GH	Growth hormone
IGF1R	Insulin-like growth factor 1 receptor
PP	Pancreatic polypeptide
DAG	Diacylglycerol
BCAAs	Branched-chain amino acids
GDH	Glutamate dehydrogenase
α_2_AAR	α_2_A-adrenergic receptor
ATPβ	ATP synthase β subunit
GK	Glucokinase
IRS1	Insulin receptor substrate 1
GLP-1	Glucagon-like peptide-1
GIP	Glucose-dependent insulinotropic polypeptide
FXR	Farnesoid X receptor
TGR5	Takeda G protein-coupled receptor 5
AdipoR	Adiponectin receptor
RBP4	Retinol-binding protein 4
miRNAs	MicroRNAs
EVs	Extracellular vesicles
TNF-α	Tumor necrosis factor-α
IL-6	Interleukin-6
IL-1β	Interleukin-1β
PC	Phosphatidylcholine
PE	Phosphatidylethanolamine
CEPT1	Choline/ethanolamine phosphotransferase 1
SIBO	Small intestinal bacterial overgrowth
LPS	Lipopolysaccharide
IRS	Insulin receptor substrate
GSK3β	Glycogen synthase kinase 3β
GCK	Glucokinase
G6P	Glucose-6-phosphate
FFAs	Free fatty acids
IGFBPs	Insulin-like growth factor binding proteins
hPL	Human placental prolactin
PGH	Placental growth hormone
SOCS3	Cytokine signal suppressor 3
SWIFT	Study of Women, Infant Feeding, and Type 2 Diabetes After GDM Pregnancy
MODY	Maturity-onset diabetes of the young
HNF1A	Hepatocyte nuclear factor 1-alpha
HNF4A	Hepatocyte nuclear factor 4-alpha
PNDM	Permanent neonatal diabetes mellitus
G6PT	Glucose-6-phosphate transporter
CRP	C-reactive protein
GSIS	Glucose-stimulated insulin secretion
VLDL	Very low-density lipoprotein
BAT	Brown adipose tissue
Adpn/Lep	Adiponectin/leptin ratio
SGLT2i	SGLT2 inhibitors
GLP-1RA	GLP-1 receptor agonist
AAV	Adeno-associated virus
